# Antinuclear Antibodies With a Nucleolar Pattern Are Associated With a Significant Reduction in the Overall Survival of Patients With Leukemia: A Retrospective Cohort Study

**DOI:** 10.3389/fonc.2021.631038

**Published:** 2021-02-26

**Authors:** Rong Wang, Huijuan Zhao, Yang Liu, Bing Kang, Jun Cai

**Affiliations:** ^1^ Department of Clinical Laboratory, Henan Provincial People’s Hospital, People’s Hospital of Zhengzhou University, Zhengzhou, China; ^2^ Basic Medical College, Henan University of Science and Technology, Luoyang, China; ^3^ Institute of Medical Genetics, Henan Provincial People’s Hospital, Henan Provincial People’s Hospital, People’s Hospital of Zhengzhou University, Zhengzhou, China

**Keywords:** antinuclear antibody, the nucleolar pattern, leukemia, cohort, overall survival

## Abstract

**Objective:**

Antinuclear antibodies (ANAs) have been reported to be associated with cancers. However, the role of different ANA patterns in cancers is poorly understood, especially in leukemia. This study aimed to investigate the association between ANA patterns and the outcome of leukemia in a retrospective cohort.

**Methods:**

A total of 429 adult patients initially diagnosed with leukemia at Henan Provincial People’s Hospital from January 2014 to December 2018 were included in this study, including information on patients without positive ANAs at the time of initial diagnosis, preexisting autoimmune diseases, infectious diseases, etc. The data were retrieved up to December 2020. The final sample included 196 adult patients. The risk of death outcome according to ANA patterns was estimated using multivariable Cox proportional hazards models and the overall survival for ANA patterns was analyzed using Kaplan–Meier curve.

**Results:**

ANAs with a nucleolar pattern *versus* negative ANA were associated with a two-fold increased risk of death outcome in leukemia, independent of sex, age, leukemia immunophenotype, cytogenetic abnormality, treatment, and blood transfusion. Further analysis revealed that the association was more significant in elder patients (≥60 years) and patients treated with tyrosine kinase inhibitor or chemotherapy (P for interaction = 0.042 and 0.010). Notably, the patients with a nucleolar pattern had shorter survival than the patients with a non-nucleolar pattern or without ANA (*p* < 0.001).

**Conclusion:**

ANAs with a nucleolar pattern are a significant predictor of poor prognosis, providing clues for prognostic assessment in patients with leukemia.

## Introduction

The development of autoantibodies is a consequence of the breakdown of immunological tolerance, and the autoantibodies are generated in the context of diseases other than autoimmune disorders, such as cancers (1, 2). Antinuclear antibodies (ANAs) are a spectrum of autoantibodies that react with various nuclear and cytoplasmic components of normal human cells (3). They are very useful as serological markers for different autoimmune diseases; recent studies have shown that ANAs are presented, not only in autoimmune diseases, but also in the serum of patients with different types of cancers (4–7). First, autoantibodies are reported to participate in carcinogenesis (8), and ANA titers are related to the early detection of many tumors (9, 10). Autoantibodies have been reported to have high sensitivity and specificity for the diagnosis of breast and other cancers (11, 12). Additionally, positive ANAs in malignancies are associated with cancer prognosis (10, 13). The appearance of autoantibodies or clinical manifestations of autoimmunity during treatment with interferon alfa-2b is associated with statistically significant improvements in relapse-free survival and overall survival in patients with melanoma (4). Furthermore, the presence of the examined preexisting ANAs is associated with clinical benefit and the development of immune-related adverse events in patients with non-small cell lung cancer treated with nivolumab or pembrolizumab (5).

In indirect immunofluorescence, ANAs can display different nuclear patterns depending on the targeted antigens; these patterns have been recently defined by the International Consensus on ANA Patterns (ICAP) (14). Antinuclear autoantibodies include AC-1 to AC-14, while anti-cytoplasmic autoantibodies include AC-15 to AC-23, besides the mitotic patterns (AC-24 to AC-28). Furthermore, different ANA patterns have been associated with the presence of cancer. ANAs with a homogeneous and speckled immunofluorescence pattern are associated with lack of cancer, while those with a nucleolar pattern are associated with the presence of cancer (15). The positivity for the anti-centromere antibody is reported as a statistically significant risk factor for cancer (16).

Despite the close relationship of ANAs with cancers, the clinical value of ANAs, especially the ANA patterns, in leukemia, is poorly understood. This retrospective study aimed to investigate the association between the presence of ANAs, especially the ANA patterns, and the outcome of leukemia. The findings revealed that ANAs with a nucleolar pattern were predictive of poor prognosis, thus providing some clues for the prognostic assessment in patients with leukemia.

## Materials and Methods

### Study Design and Patients

**Figure 1:**
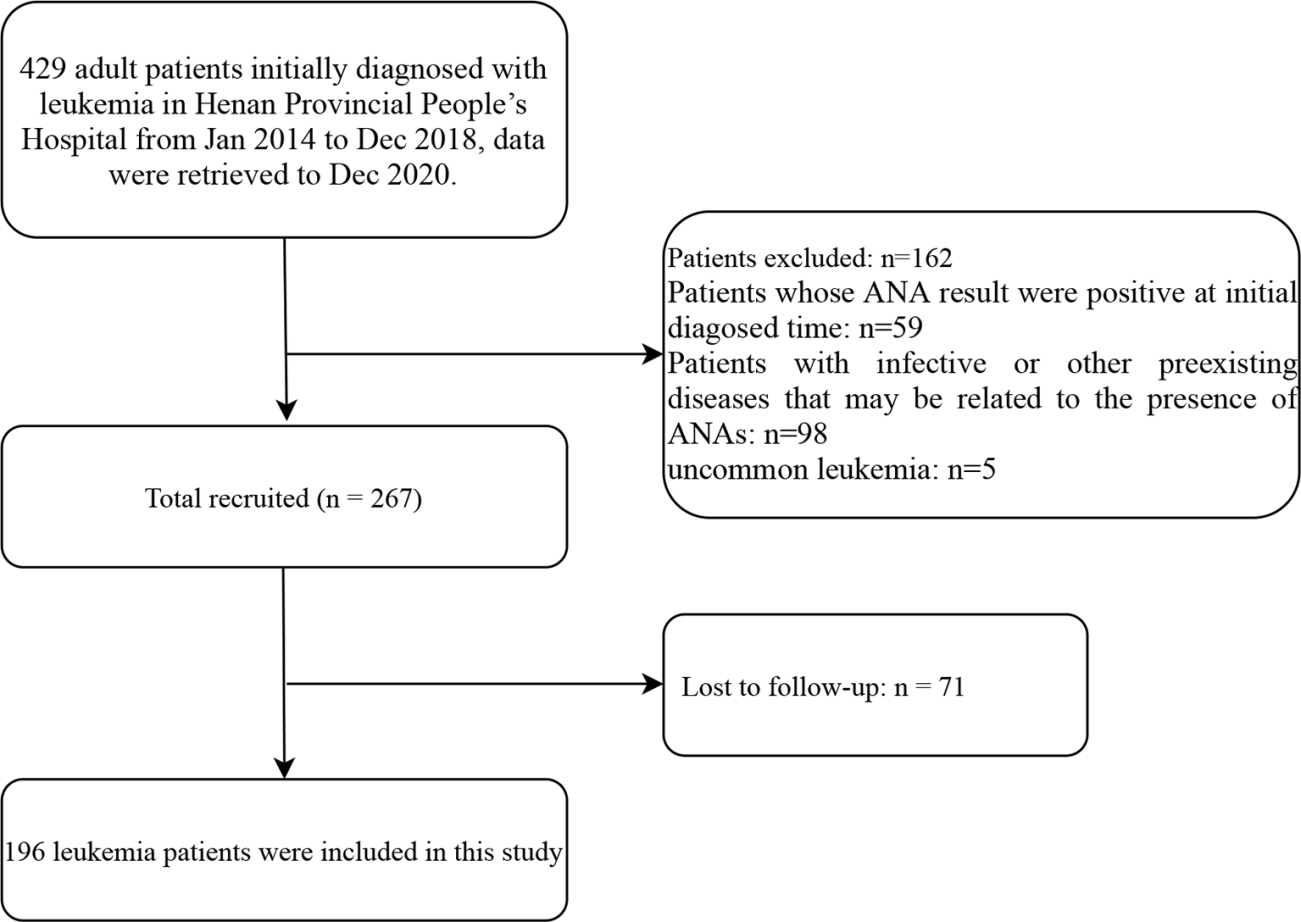
Flow diagram of the screening and enrollment of study patients. ANA, antinuclear antibodies.

This was a population-based retrospective cohort study conducted at Henan Provincial People’s Hospital (Zhengzhou, China). A total of 429 adult patients initially diagnosed with leukemia from January 2014 to December 2018 were included in this study. The data were retrieved up to December 2020. All patients were diagnosed according to the guidance of the National Comprehensive Cancer Network. The exclusion criteria were as follows: patients whose ANA results were positive at the time of initial diagnosis (n = 59), and patients diagnosed with infective diseases, autoimmune diseases and other preexisting diseases that might be related to the presence of ANAs (n = 98) and uncommon leukemia (n = 5). Furthermore, another 71 patients were excluded because of missing follow-up data. The resulting cohort included 196 patients for the final analysis (**Figure 1**). In this study, the ANA results were analyzed at least 2 months after treatment in the patients with leukemia. Notably, the ANA results in patients treated with allogeneic hematopoietic hematopoietic stem cell transplantation (allo-HSCT) were negative before HSCT, and in these patients, only the record of blood transfusion history after they were treated with allo-HSCT were obtained. The non-HSCT treatments (*e.g.* chemotherapy) mentioned in this study referred to the early treatment or induction therapy. These treatments included tyrosine kinase inhibitor (TKI) in chronic myeloid leukemia (CML), FCR/FC (F: fludarabine, C: cyclophosphamide, R: rituximab) in chronic lymphocytic leukemia (CLL), VDCLP (V: vincristine, D: daunorubicin, C: cyclophosphamide, L: L-asparaginase, P: prednisone) and VDCLP + TKI in acute lymphocytic leukemia (ALL), all-trans retinoic acid (ATRA) + arsenic trioxide in acute promyelocytic leukemia (APL, M3), cytarabine+ idarubicin (IDA)/daunorubicin (DNR) in acute myeloid leukemia (AML, not APL) according to corresponding guidelines standardly (17–21). These substances are representatives of each kind of drugs. The preconditioning regimen of allo-HSCT was performed according to the modified Bu/Cy+ATG regimen (Bu: busulphan, Cy: cyclophosphamide, ATG: anti-thymocyte globulin) in the Consensus of Allogeneic Hematopoietic Transplantation for Hematological Diseases in China (2014)-Indication, Conditioning Regimen and Donor Selection (22). Additionally, the stem cells were obtained from peripheral blood and bone marrow according to the guideline standardly (22). The information on age, sex, leukemia immunophenotype, cytogenetic abnormality, treatment and blood transfusion history was obtained from the electronic medical record system. This study was conducted under the guidance of the Ethics Committee of Henan Provincial People’s Hospital and written informed consent was obtained from all patients of the study.

### ANA Detection

Indirect immunofluorescence kits were used for ANA detection following the manufacturer’s protocols (Oumeng, Germany). ANA detection was performed by using the substrate slides of human epithelial type 2 cell line) and monkey liver tissue, along with fluorescein isothiocyanate anti-human IgG conjugate. The serums were titered from 1/100 to the endpoint, and the results were expressed as the last positive dilution. The ANA titers more than 1:100 (≥1:100) were considered as a positive result in this study. The ANA patterns included nuclear speckled (AC-2, -4 and -5), nuclear homogeneous (AC-1), nucleolar (AC-8, -9 and -10), cytoplasmic speckled (AC-18, -19 and -20), and other kinds of patterns (nuclear envelope (AC-11 and -12), discrete nuclear dots (AC-6 and -7), centromere pattern (AC-3), rods and rings (AC-23), etc.). These patterns were recently defined by the ICAP (14).

### Statistical Analysis

Continuous variables were presented as mean ± standard deviation (SD) or medians (interquartile range 25–75% [IQR]), whereas categorical variables were indicated as frequencies (%). For demographic and clinical characteristics analysis, the statistical differences between the ANA-negative group and ANA-positive group were tested with the unpaired Student’s t-test or Mann–Whitney *U*-test depending on the normality of the distribution for continuous variables, and chi-square test for categorical variables.

Hazard ratios (HRs) and 95% confidence intervals (CIs) were calculated for leukemia outcome with variables using Cox proportional hazards models. Both non-adjusted and multivariate-adjusted models were used to evaluate the association between ANA pattern and the leukemia outcome. The crude model was the non-adjusted model with no covariates adjusted. The adjusted I model was the minimally-adjusted model with only sociodemographic variables adjusted including sex and age. The adjusted II model was adjusted with covariates (age, sex, leukemia immunophenotype, and cytogenetic abnormality). The adjusted III model was the fully-adjusted model with all covariates adjusted (age, sex, leukemia immunophenotype, cytogenetic abnormality, treatment, and blood transfusion). The subgroup analyses were performed using stratified Cox regression models. Age, which is a continuous variable, we first converted it into a categorical variable according to the mean (45 years) or the clinical significance (60 years), and then performed an interaction test. For leukemia immunophenotype, the immunophenotypes of CML, M3 (AML) and CLL were classified into the non-progressive group, while the immunophenotypes of M2, M4, M5 in AML, and ALL were classified into the progressive group according to the result of univariate analysis and clinical significance. For leukemia treatment, the treatment I included TKI, FCR/FC, and ATRA + arsenic trioxide; treatment II included VDCLP + TKI, VDCLP and Ara-c + DA/DNR; and treatment III included allo-HSCT. The interaction across subgroups was tested using the likelihood ratio test. The overall survival curve was assessed using the Kaplan–Meier (K-M) method.

SPSS 17.0 (Chicago, IL, USA) and the statistical software packages R version 3.4.3 (The R Foundation, Vienna, Austria) were used for analysis. A two-sided *P* value <0.05 was considered to be statistically significant.

## Results

### Demographic and Clinical Characteristics of the Patients With Leukemia

Among the 196 patients included in the study, the outcome of 56 cases was death, while 140 cases survived with a median 21-month follow-up. The demographic and clinical characteristics of the patients are shown in **Table 1**. Almost half of the patients (108, 55.1%) were male, and the mean age of all patients was 45.6 ± 16.7 years. No significant difference was found in the positive rates of ANAs among leukemia immunophenotype, cytogenetic abnormality, treatment and blood transfusion. The positive rates of ANAs in patients with death outcome were significantly higher than those in surviving patients (*p* = 0.044), suggesting that the presence of ANAs was associated with the outcome of patients with leukemia.

**Table 1:** Demographic and clinical characteristics of patients with leukemia.

Variables	Total patients(n = 196)	ANA Negative (n = 128)	ANA Positive (n = 68)	P value
Sex, n (%)				0.77
Male	108 (55.1)	72 (56.2)	36 (52.9)	
Female	88 (44.9)	56 (43.8)	32 (47.1)	
Age, year, Mean ± SD	45.6 ± 16.7	45.7 ± 16.3	45.6 ± 17.4	0.962
Leukemia immunophenotype, n (%)				0.854
CML	32 (16.3)	23 (18)	9 (13.2)	
CLL	14 ( 7.1)	10 (7.8)	4 (5.9)	
ALL	51 (26.0)	32 (25)	19 (27.9)	
M2	53 (27.0)	33 (25.8)	20 (29.4)	
M3	17 ( 8.7)	13 (10.2)	4 (5.9)	
M4	13 ( 6.6)	8 (6.2)	5 (7.4)	
M5	16 ( 8.2)	9 (7)	7 (10.3)	
Cytogenetic abnormality, n (%)				0.983
Negative	105 (53.6)	68 (53.1)	37 (54.4)	
Positive	91 (46.4)	60 (46.9)	31 (45.6)	
Treatment, n (%)				0.082
TKI	30 (15.3)	23 (18)	7 (10.3)	
FCR/FC	14 ( 7.1)	10 (7.8)	4 (5.9)	
VDCLP+TKI	13 ( 6.6)	9 (7)	4 (5.9)	
VDCLP	14 ( 7.1)	10 (7.8)	4 (5.9)	
ATRA+arsenic trioxide	17 ( 8.7)	13 (10.2)	4 (5.9)	
Ara-c+IDA/DNR	56 (28.6)	39 (30.5)	17 (25)	
allo-HSCT	52 (26.5)	24 (18.8)	28 (41.2)	
Blood transfusion, n (%)				1
No	39 (19.9)	25 (19.5)	14 (20.6)	
Yes	157 (80.1)	103 (80.5)	54 (79.4)	
ANA.pattern, n (%)				<0.001
Negative	128 (65.3)	128 (100)	0 (0)	
Nuclear speckled pattern	21 (10.7)	0 (0)	21 (30.9)	
Cytoplasmic speckled pattern	12 ( 6.1)	0 (0)	12 (17.6)	
Nucleolar pattern	25 (12.8)	0 (0)	25 (36.8)	
Other patterns	10 ( 5.1)	0 (0)	10 (14.7)	
Outcome, n (%)				0.044
Survive	140 (71.4)	98 (76.6)	42 (61.8)	
Death	56 (28.6)	30 (23.4)	26 (38.2)	
Overall survival, month, Median (IQR)	21.0 (10.0,39.2)	20.0 (11.0,36.5)	25.0 (7.8,43.5)	0.449

ANA, antinuclear antibodies; SD, standard deviation; CML, chronic myelogenous leukemia; CLL, chronic lymphocytic leukemia; ALL, acute lymphoblastic leukemia; the immunophenotypes of M2, M3, M4 and M5 are acute myelogenous leukemia; TKI, tyrosine kinase inhibitor, FCR/FC (F, fludarabine; C, cyclophosphamide; R, rituximab); VDCLP (V, vincristine; D, daunorubicin; C, cyclophosphamide; L, L-asparaginase; P, prednisone); ATRA, all-trans retinoic acid; Ara-c + IDA/DNR (cytarabine + idarubicin/daunorubicin); allo-HSCT, allogeneic hematopoietic hematopoietic stem cell transplantation; IQR, interquartile range.

The aforementioned substances are representatives of each kind of drugs. The other patterns of ANA include nuclear homogeneous, peripheral pattern, etc.; P < 0.05 indicated a statistically significant difference.

### Univariate Cox Regression Models in the Leukemia Patients

**Table 2** shows the HRs and 95% CIs for the risk of death outcome in patients with leukemia by different variables. The results indicated that the female patients were associated with a 45% reduced risk of death outcome referenced to the male patients (95% CIs 0.31, 0.96). Additionally, the patients diagnosed with ALL, M2, M4 and M5 had a higher risk of death outcome compared with those diagnosed with CML. The patients treated with VDCLP + TKI, VDCLP, Ara-c + IDA/DNR, and allo-HSCT had a higher risk of death outcome referenced to those treated with TKI. Meanwhile, the patients with blood transfusion history were associated with a 3.62-fold increased risk of death outcome referenced to those without blood transfusion history (95% CIs 1.66, 12.88). Furthermore, the patients with a nucleolar pattern in ANA had a 1.99-fold increased risk of death outcome compared with those with negative ANA (95% CIs 1.62, 5.51).

**Table 2:** Univariate Cox regression models in patients with leukemia.

Variables	HR (95% CI)	*P* value
Sex, n (%)		
Male	Ref	
Female	0.55 (0.31,0.96)	0.034
Age, year, Mean ± SD	1.0064 (0.991,1.022)	0.418
Leukemia immunophenotype, n (%)		
CML	Ref	
CLL	3.83 (0.69,21.24)	0.124
ALL	11.21 (2.53,49.65)	0.001
M2	9.05 (2.05,39.98)	0.004
M3	0 (0,Inf)	0.997
M4	17.09 (3.48,83.98)	<0.001
M5	14.01 (2.75,71.5)	0.001
Cytogenetic abnormality, n (%)		
Negative	Ref	
Positive	0.88 (0.52,1.5)	0.644
Treatment, n (%)		
TKI	Ref	
FCR/FC	3.52 (0.64,19.43)	0.149
VDCLP+TKI	14.32 (2.78,73.81)	0.001
VDCLP	17.28 (3.5,85.3)	<0.001
ATRA+arsenic trioxide	0 (0,Inf)	0.997
Ara-c+IDA/DNR	14.04 (3.2,61.55)	<0.001
allo-HSCT	5.49 (1.22,24.79)	0.027
Blood transfusion, n (%)		
No	Ref	
Yes	4.62 (1.66,12.88)	0.003
ANA.pattern, n (%)		
Negative	Ref	
Nuclear speckled pattern	0.91 (0.35,2.36)	0.848
Cytoplasmic speckled pattern	0.77 (0.18,3.23)	0.72
Nucleolar pattern	2.99 (1.62,5.51)	<0.001
Other patterns	1.04 (0.32,3.43)	0.945

Inf, infinity; Ref, reference.

Data presented are HRs and 95% CIs. P < 0.05 was considered statistically significant.

### Association of ANA Patterns With the Outcome of Leukemia Patients

**Table 3** shows the HRs and 95% CIs for the risk of death outcome by ANA patterns. Considering the above result that there was no significant association between the nuclear speckled, cytoplasmic speckled, and other kinds of patterns, with the outcome in patients with leukemia, we reclassified the nuclear speckled, cytoplasmic speckled and other kinds of patterns, as non-nucleolar pattern. In the non-adjusted model, a 1.99-fold increased risk of leukemia death was observed in ANAs with a nucleolar pattern compared with negative ANA (*P* for trend = 0.003). After adjustment for age and sex, the HRs were 3.02 (95% CIs 1.62, 5.62, P for trend = 0.003). After adjustment for age, sex, leukemia immunophenotype and cytogenetic abnormality, the HRs were 2.41 (95% CIs 1.25, 4.66, P for trend = 0.031). Furthermore, after adjustment for age, sex, leukemia immunophenotype, cytogenetic abnormality, treatment and blood transfusion, the HRs were 3.65 (95% CIs 1.78, 7.52, P for trend = 0.001).

**Table 3:** Multivariable Cox regression models evaluating the association between ANA patterns and the outcome of patients with leukemia.

Variables	N. (%)	Crude HR (95% CI)	Adjusted Ⅰ HR (95% CI)	Adjusted Ⅱ HR (95% CI)	Adjusted Ⅲ HR (95% CI)
ANA pattern	196				
Negative	128 (65.3%)	Ref			
Non-nucleolar	43 (21.9%)	0.91 (0.44,1.87)	0.98 (0.48,2.02)	0.82 (0.39,1.72)	1.14 (0.52,2.51)
Nucleolar	25 (12.8%)	2.99 (1.62,5.51)	3.02 (1.62,5.62)	2.41 (1.25,4.66)	3.65 (1.78,7.52)
P for trend		0.003	0.003	0.031	0.001

Data presented are HRs and 95% CIs. Adjusted model Ⅰ adjusted for age and sex. Adjusted model Ⅱ adjusted for age, sex, leukemia immunophenotype and cytogenetic abnormality. Adjusted model Ⅲ adjusted for age, sex, leukemia immunophenotype, cytogenetic abnormality, treatment and blood transfusion. The non-nucleolar patterns of ANAs included the nuclear speckled pattern, cytoplasmic speckled and other kinds of patterns. P < 0.05 was considered statistically significant.

### Subgroup Analyses

Stratified and interactive analyses were performed to detect whether the association between ANA patterns and the outcome of leukemia was stable in different subgroups (**Table 4**). Age, which is a continuous variable, was converted into a categorical variable according to the mean (45 years) or the clinical significance (60 years). For leukemia immunophenotype, the immunophenotype of CML, M3 (AML) and CLL were classified into the non-progressive group, while the immunophenotypes of M2, M4, M5 in AML, and ALL were classified into the progressive group according to the result of univariate analysis and clinical significance. Furthermore, the treatment of TKI, FCR/FC, ATRA + arsenic trioxide were classified into treatment I; VDCLP + TKI, VDCLP, Ara-c + IDA/DNR into treatment II; and the allo-HSCT treatment into treatment III based on the univariate analysis result and clinical significance.

**Table 4:** Subgroup analyses of the association between ANA pattern and the outcome of patients with leukemia.

Subgroup	ANA pattern			P for trend	P for interaction
	Negative	Non-nucleolar	Nucleolar		
Sex					0.569
Male	1(Ref)	1.25 (0.46,3.43)	3.05 (1.33,7)	0.012	
Female	1(Re)	0.78 (0.23,2.64)	4.77 (1.27,17.93)	0.09	
Age I					0.171
<45	1(Ref)	0.32 (0.04,2.73)	3.65 (1.13,11.78)	0.063	
≥45	1(Re)	1.31 (0.54,3.2)	3.32 (1.4,7.87)	0.01	
Age II					
<60	1(Ref)	0.53 (0.18,1.62)	2.81 (1.33,5.94)	0.02	0.042
≥60	1(Ref)	6.22 (0.98,39.35)	22.28 (2.4,206.63)	0.005	
Leukemia immunophenotype					0.168
Non-progressive	1(Ref)	35.18 (1.2,1034.3)	106.7 (2.6,4385.92)	0.008	
Progressive	1(Ref)	0.77 (0.33,1.8)	2.71 (1.31,5.59)	0.018	
Treatment					
I	1(Ref)	35.18 (1.2,1034.3)	106.7 (2.6,4385.92)	0.008	0.010
II	1(Ref)	1.15 (0.4,3.34)	6.11 (2.56,14.61)	<0.001	
III	1(Ref)	0.2 (0.04,1.05)	0.63 (0.17,2.36)	0.387	
Blood transfusion					0.505
No	1(Ref)	0.76 (0.02,34.72)	53.67 (0,Inf)	0.886	
Yes	1(Ref)	0.94 (0. 4,2.19)	3.69 (1.79,7.58)	0.001	

Data presented are HRs and 95% CIs. Inf: Infinity; Ref: reference. The subgroup in age Ⅰ was analyzed according to the mean of age, and the subgroup in age Ⅱ was analyzed according to the clinical significance. The non-progressive leukemia immunophenotypes included the immunophenotype of CML, M3 (AML) and CLL, while the progressive leukemia immunophenotypes included the immunophenotypes of M2, M4, M5 in AML, and ALL. Treatment Ⅰ included TKI, FCR/FC, ATRA + arsenic trioxide. Treatment Ⅱ included VDCLP + TKI, VDCLP, Ara-c + IDA/DNR. Treatment Ⅲ included allo-HSCT. The subgroup analyses were adjusted with covariates except the analyzed variable. P < 0.05 was considered statistically significant.

The data showed that the associations with the nucleolar pattern of ANAs were stable across sexes (*P* for interaction = 0.569) and leukemia immunophenotypes (*P* for interaction = 0.168). Although the results that the associations with the nucleolar pattern of ANAs were stable across age obtained using different subgroup analysis were similar, the data showed that the associations with the nucleolar pattern of ANAs were stronger for the elderly patients (≥60 years) (HR 22.28, 95% CIs 2.4, 206.63), indicating that age played an interactive role in the association between ANA patterns and the death outcome of leukemia (*P* for interaction = 0.042). Furthermore, the data suggested that the treatment played an interactive role in the association between ANA patterns and the death outcome of leukemia (*P* for interaction = 0.010). The associations with the nucleolar pattern of ANAs were stronger for the patients treated with TKI or chemotherapy (treatment I and II), while no significant associations were observed in those treated with allo-HSCT (treatment III). Additionally, the associations with the nucleolar pattern of ANAs were stronger for the patients with blood transfusion history (HR 3.69, 95% CIs 1.79, 7.58), and no significant associations were observed in the patients without blood transfusion history.

### Overall Survival Analysis

**Figure 2:**
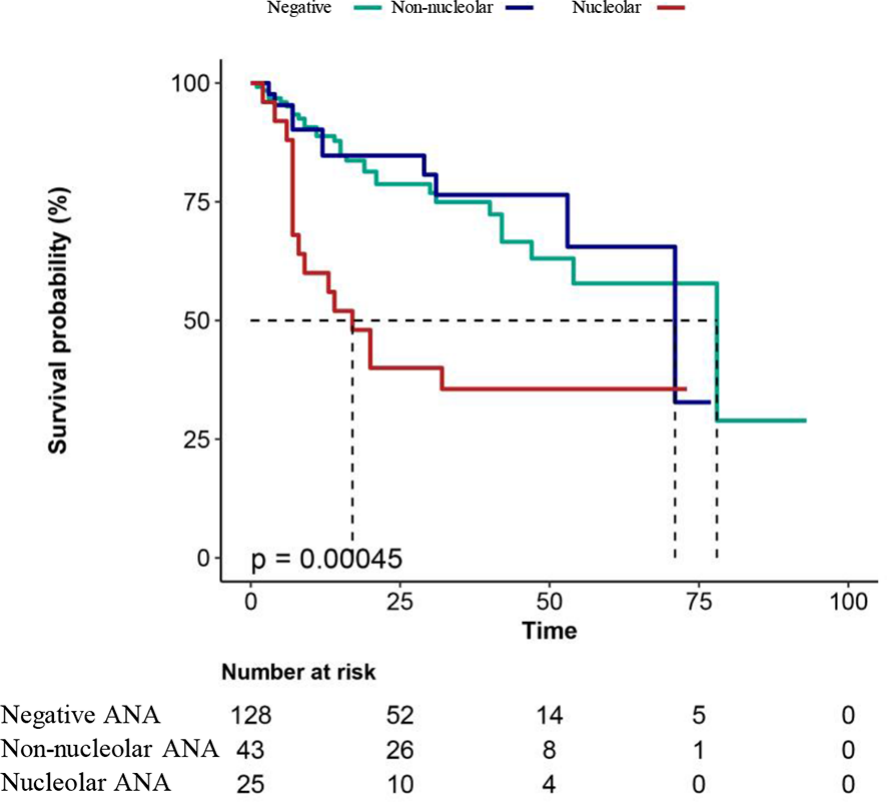
Overall survival analysis in patients with leukemia. Negative, patients with leukemia without ANA; non-nucleolar, patients with non-nucleolar ANAs; nucleolar, patients with nucleolar ANAs. A two-sided P value < 0.05 indicated a statistically significant difference.

K–M curve was used to investigate the association between ANA patterns and the overall survival time in patients with leukemia, as shown in **Figure 2**. The results showed that the patients with a nucleolar pattern had shorter survival compared with the patients with a non-nucleolar pattern or without ANA (*p* < 0.001), suggesting that the presence of the nucleolar pattern was associated with a statistically significant reduction in the overall survival of patients with leukemia.

## Discussion

In this population-based cohort study, patients whose ANA results were positive at initial diagnosis were excluded. The ANA results in this study were analyzed at least 2 months after treatment in the patients with leukemia. Therefore, the ANAs assessed in this study were therapy-induced ANAs. Interestingly, ANAs with a nucleolar pattern were found to be associated with poor prognosis in patients, independent of age, sex, leukemia immunophenotype, cytogenetic abnormality, treatment and blood transfusion. The study further revealed that the associations with the nucleolar pattern of ANAs were stronger for the elderly patients (≥60 years). Additionally, a significant association was observed in patients treated with TKI or chemotherapy, rather than in those treated with allo-HSCT. Notably, patients with a nucleolar pattern had shorter survival time compared with those with a non-nucleolar pattern and without ANA.

Most previous studies reported that the appearance of ANAs was associated with improved outcome in patients with cancer (3, 4, 23), possibly due to more vigorous activation of the immune system fighting against malignant cells (10, 13, 24). Despite contradictory reports, the presence of ANAs might result in a poor prognosis in patients treated with combinatorial immunotherapy and chemotherapy (25–27). Consistent with the aforementioned findings, the appearance of ANAs was associated with a poor prognosis in patients with leukemia in this study. Previous studies showed that these autoantibodies had a wide spectrum of action, from mimicking the stimulation of hormone-dependent receptors, blocking neural transmission, inducing altered signaling and inflammation, to triggering cell lysis and neutrophil activation (28–30). The difference in immune response might account for the controversy and hence needs further investigation.

Additionally, the significance of the ANA patterns is ignored in clinical practice and research. A recent study showed that the presence of ANAs with a nucleolar pattern was associated with an increased relative risk of cancer (15). Furthermore, the significance of ANA patterns, especially the cell-cycle related ANAs, was emphasized in carcinoma (31, 32). Interestingly, the ANAs with a nucleolar pattern were associated with a significant reduction in the overall survival of patients with leukemia in this study, suggesting the clinical significance of ANA patterns in malignancies. The expression of mutated proteins, which show a nucleolar pattern when targeted by ANA during the cancer treatment, is one possible mechanism (33). The nucleophosmin and nucleolin, which are mutated nucleolar proteins, are reported to be commonly overexpressed in cancer cells and influences the cell survival, proliferation and invasion, by acting on different cellular pathways (34, 35).

The present subgroup analysis revealed that the association between ANA patterns and the death outcome of leukemia stably existed between the layers except treatment regimens and blood transfusion history. It showed that a significant association was observed in patients treated with TKI or chemotherapy, rather than in those treated with allo-HSCT (*P* for trend <0.01). An increasing number of clinical reports have shown an association between transplantation and humoral autoimmunity, and transplantation can act as a trigger for the development of autoantibodies (36). The present study found that patients treated with allo-HSCT had higher rates of ANAs compared with patients treated with TKI or chemotherapy, which was similar to previous studies showing that the patients treated with HSCT had a significantly higher prevalence of ANAs compared with healthy controls (37). Additionally, the appearance of ANAs is considered as a sign of chronic graft-versus-host disease (cGVHD) in patients treated with HSCT. However, cGVHD activity or severity and the overall survival were reported to be not associated with the presence of ANAs (37), indicating that the GVHD may have an impact on the leukemia outcome, rather than the presence of ANA. The present study focused on the association between the ANA patterns and leukemia outcome in patients with different treatment regimens. Similarly, the associations between ANA patterns and leukemia outcome were not observed in patients treated with allo-HSCT, though the presence of GVHD was not analyzed in this study, suggesting that ANA patterns had limited value in predicting the outcome in patients treated with allo-HSCT.

The present study also showed that a significant association was observed in patients with blood transfusion history, rather than in those without blood transfusion history (*P* for trend = 0.001). Patients exposed to multiple rounds of blood transfusion tend to develop autoantibodies. The frequency of ANAs in multi-transfused patients with thalassemia major was higher than that in age- and sex- matched healthy controls (38). The increased ANA production might be associated with transfusion-induced increases in CD5 B cells (39), which needs further investigation.

This study had certain limitations. First, cytogenetic abnormalities constitute an important factor for leukemia diagnosis, treatment, and prognosis. Although the cytogenetic abnormalities were included, this study only analyzed the association between the presence of cytogenetic abnormalities and ANAs; the association between different cytogenetic abnormalities and the leukemia outcome was not explored. Second, this subgroup analysis revealed that the associations with the nucleolar pattern of ANAs were stronger for the elderly patients (≥60 years) (HR 22.28, 95% CIs 2.4, 206.63, *P* for interaction = 0.042). This study lacked data on the baseline examination, including anthropometric measurements (weight, waist circumference, and blood pressure) and a questionnaire on the lifestyle characteristics, including physical activity, smoking, and alcohol habits, which were closely associated with age. Furthermore, an increased prevalence of ANA was observed in the old individuals (40). All referred factors might have caused a potential bias in the results. Third, the ANA patterns were recently defined by the ICAP (14), and the anti-nucleolar autoantibodies can be homogeneous (AC-8), clumpy (AC-9), or punctate (AC-10), which have different target antigens. Additionally, the target antigens of Ku, NOR-90, PM/Scl-100, PM/Scl-75, RNAP-III, Scl-70, etc., are known to possibly elicit a nucleolar ANA pattern. However, the different target antigens of the nucleolar pattern were not involved in our study, suggesting that different antigens of nucleolar ANA pattern might have different relationships with the prognosis in patients with leukemia. Additionally, although the patients initially diagnosed with leukemia and autoimmune diseases were excluded in this study, whether the patients with ANA positivity after treatment had symptoms of autoimmunity, was not recorded in the follow-up, thus we couldn’t investigate further into the relationship between leukemia and autoimmunity disease.

In conclusion, ANAs with a nucleolar pattern were independently associated with an increased risk of death outcome in patients with leukemia in this retrospective cohort study. Additionally, the patients with a nucleolar pattern had shorter survival compared with the patients with a non-nucleolar pattern or without ANA, suggesting ANAs with a nucleolar pattern could serve as a predictor of poor prognosis in patients with leukemia. The findings emphasized the clinical significance of the ANA patterns in the prognosis of malignancies. Further studies are needed to explore which particular antibody against nuclear antigens is responsible for the association, and the potential mechanisms.

## Data Availability Statement

The original contributions presented in the study are included in the article/supplementary material. Further inquiries can be directed to the corresponding authors.

## Ethics Statement

The studies involving human participants were reviewed and approved by Ethics Committee of Henan Provincial People’s Hospital. The patients/participants provided their written informed consent to participate in this study. Written informed consent was obtained from the individual(s) for the publication of any potentially identifiable images or data included in this article.

## Author Contributions

RW and JC conceived and designed the study. YL and BK conducted the clinical data extraction and literature search. RW and HZ conducted experiment and data analysis. RW and JC wrote the draft of the manuscript. YL, JC, BK, and HZ critically revised the manuscript. All authors contributed to the article and approved the submitted version.

## Funding

This work was supported by grants 82002210 from the National Natural Science Foundation of China (NSFC).

## Conflict of Interest

The authors declare that the research was conducted in the absence of any commercial or financial relationships that could be construed as a potential conflict of interest.
